# High‐grade B‐cell lymphoma with *MYC* and *BCL2* rearrangements with abundant multinucleated giant tumor cells

**DOI:** 10.1002/jha2.653

**Published:** 2023-02-13

**Authors:** Man Zhang, Yu Yang

**Affiliations:** ^1^ Department of Pathology, Immunology and Laboratory Medicine University of Florida College of Medicine Gainesville Florida USA

**Keywords:** FISH, immunophenotyping, lymphomas

1

A 75‐year‐old female presented with a mass on the left side of the neck. A positron emission tomography scan showed an enlarged left cervical lymph node (2.6 cm) with a standard uptake value of 22.05. Fine needle aspiration was initially performed and revealed clusters of cohesive malignant cells that were suspicious for carcinoma. An excisional biopsy later showed the lymph node was effaced by an atypical lymphoid infiltrate composed of sheets of very pleomorphic abnormal cells with dense chromatin admixed with frequent multinucleated giant tumor cells (Figure 1 upper row; hematoxylin and eosin stain, ×400 [left], ×1000 [right]). By immunohistochemistry, the neoplastic cells including the giant forms are diffusely and strongly positive for CD20 (Figure 1 lower left; ×400), PAX5 (Figure 1 lower right; ×400), CD30, and MUM1 and negative for CD10, CD15, Perforin, ALK, cyclin D1, SOX11, CD21, CD23, CD34, TdT, human herpesvirus‐8 (HHV‐8), CD138, Cytokeratin, Epithelial membrane antigen (EMA), and S100. The additional Fluorescence In Situ Hybridization (FISH) analysis demonstrated *MYC* and *BCL2* rearrangements and is consistent with high‐grade B‐cell lymphoma with *MYC* and *BCL2* rearrangements (“double‐hit” lymphoma).

**FIGURE 1:**
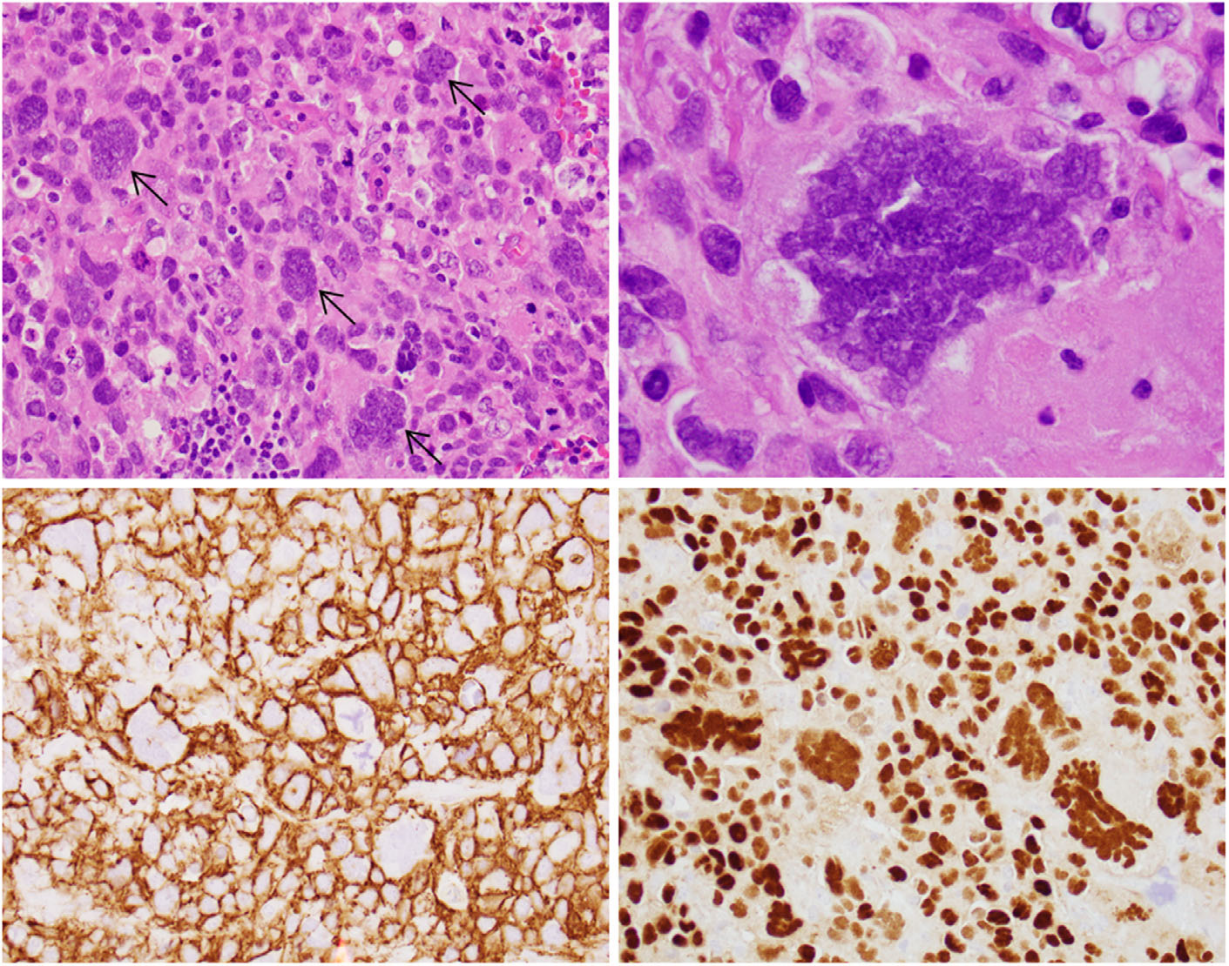
Upper row; lymph node biopsy shows sheets of pleomorphic abnormal cells with dense chromatin admixed with frequent multinucleated giant tumor cells (hematoxylin and eosin stain, ×400 [left], ×1000 [right]). Lower row; immunohistochemical (IHC) studies show that the neoplastic cells including the giant forms are diffusely and strongly positive for CD20 (left; ×400), PAX5 (right; ×400).

Multinucleated giant tumor cells are highly unusual morphologic features in non‐Hodgkin lymphoma and may be misinterpreted as nonhematopoietic malignancies by morphology alone. Therefore, awareness of this unusual morphology and including additional ancillary studies such as immunophenotyping and FISH study would be essential to achieve an accurate diagnosis.

## CONFLICT OF INTEREST STATEMENT

The authors have no conflict of interest to disclose

## ETHICS APPROVAL STATEMENT

None.

## Data Availability

The data that support the findings of this study are available from the corresponding author upon reasonable request.

